# Application of Laser-Induced Breakdown Spectroscopy Coupled With Spectral Matrix and Convolutional Neural Network for Identifying Geographical Origins of *Gentiana rigescens* Franch

**DOI:** 10.3389/frai.2021.735533

**Published:** 2021-12-10

**Authors:** Xiaolong Li, Wenwen Kong, Xiaoli Liu, Xi Zhang, Wei Wang, Rongqin Chen, Yongqi Sun, Fei Liu

**Affiliations:** ^1^ College of Biosystems Engineering and Food Science, Zhejiang University, Hangzhou, China; ^2^ College of Mathematics and Computer Science, Zhejiang A&F University, Hangzhou, China; ^3^ School of Chinese Materia Medica, Yunnan University of Chinese Medicine, Kunming, China; ^4^ Yunnan Provincial Key Laboratory of Molecular Biology for Sinomedicine, Kunming, China; ^5^ Hangzhou Landa Science and Technology Co., Ltd, Hangzhou, China; ^6^ Key Laboratory of Spectroscopy Sensing, Ministry of Agriculture and Rural Affairs, Hangzhou, China

**Keywords:** geographical origin identification, variable importance measured, convolutional neural network, spectral matrix, *Gentiana rigescens* franch

## Abstract

Accurate geographical origin identification is of great significance to ensure the quality of traditional Chinese medicine (TCM). Laser-induced breakdown spectroscopy (LIBS) was applied to achieve the fast geographical origin identification of wild *Gentiana rigescens* Franch (*G. rigescens* Franch). However, LIBS spectra with too many variables could increase the training time of models and reduce the discrimination accuracy. In order to solve the problems, we proposed two methods. One was reducing the number of variables through two consecutive variable selections. The other was transforming the spectrum into spectral matrix by spectrum segmentation and recombination. Combined with convolutional neural network (CNN), both methods could improve the accuracy of discrimination. For the underground parts of *G. rigescens* Franch, the optimal accuracy in the prediction set for the two methods was 92.19 and 94.01%, respectively. For the aerial parts, the two corresponding accuracies were the same with the value of 94.01%. Saliency map was used to explain the rationality of discriminant analysis by CNN combined with spectral matrix. The first method could provide some support for LIBS portable instrument development. The second method could offer some reference for the discriminant analysis of LIBS spectra with too many variables by the end-to-end learning of CNN. The present results demonstrated that LIBS combined with CNN was an effective tool to quickly identify the geographical origin of *G. rigescens* Franch.

## Introduction

*Gentiana rigescens* Franch is a representative medicinal plant of gentiana in south China. The main medicinal ingredients of *G. rigescens* Franch are gentianine and gentiopicri. The former has significant liver protection and stomach strengthening effects and the latter has anti-inflammatory, anti-hyperthyroidism, blood glucose raising, blood pressure lowering and antibacterial effects (Acquarelli et al., 2017). *G. rigescens* Franch has been included in the “List of Species of Wild Medicinal herbs under State Key Protection”. It is a class III protected species of wild medicinal herbs, which has been listed as one of ten important endangered medicinal plants in Yunnan province in 2002.

Plants of *G. rigescens* Franch are mainly distributed in Yunnan, Guizhou, Sichuan, etc. These regions have a wide range of elevations and temperature distribution. Due to the different geographical locations with different altitudes, biological climate, soil environment and so on, the distribution area of *G. rigescens* Franch can be divided into different suitable growth grades, including unsuitability, low suitability, moderate suitability, middle suitability and high suitability (Shen et al., 2019b). Moreover, these environmental factors will lead to the differences in the secondary metabolites of Chinese medicinal herbs, affecting multi-component coordination in exerting the multi-channel and multi-target pharmacological action (Zhao et al., 2009). Therefore, the geographical origin identification is of great significance to the quality and medicinal value of *G. rigescens* Franch.

Traditional methods of origin identification include high-performance liquid chromatography (HPLC) coupled with mass spectrometry (Badmos et al., 2020), fourier transform infrared spectroscopy (FTIR) fingerprints (Zhao et al., 2020b), combined analysis of stable isotopes and multi-elements (Zhao et al., 2020a), inductively coupled plasma-mass spectrometry (ICP-MS) (Bronzi et al., 2020) and untargeted chromatographic fingerprint coupled with data fusion and chemometrics (Shen et al., 2019a). Although these methods have high detection accuracy and sensitivity, they are destructive, time-consuming and usually involve complex operating procedures, which will not meet the rapid and efficient detection demand of the market. Thus, it is necessary to put forward a rapid and accurate method without complicated pretreatment for the identification of the geographical origin.

Laser-induced breakdown spectroscopy (LIBS) fulfils the above criteria. LIBS is an atomic spectrum that can simultaneously detect multiple elements in a sample with the capability for *in-situ* or remote real time operation. So, over the years, LIBS has been applied in the quantitative and qualitative analysis of mineral resources (Kim et al., 2018), plastic detection (Stefas et al., 2019), industrial application (Noll et al., 2018) and so on. In this study, LIBS was used to identify the geographical origins of *G. rigescens* Franch. So far, most people have used machine learning methods for LIBS spectra analysis (Liu et al., 2019), and few have used deep learning. The possible reason is that the number of variables in a LIBS spectrum is so large that it is easy to cause overfitting for deep learning. Convolutional neural network (CNN) is one kind of deep learning and has been popularly applied in various fields. CNN has shown competitive or better performances compared with machine learning methods (Liu et al., 2017; Yu et al., 2018). Therefore, CNN was taken into consideration to improve the identification efficiency in this study. To solve the problems above, we proposed two methods for LIBS spectra. One was selecting feature variables to reduce the representation learning burden of one-dimensional CNN (1D-CNN). The other was transforming the spectrum into spectral matrix by spectrum segmentation and recombination to achieve end-to-end training of two-dimensional CNN (2D-CNN). The results showed that both methods could obtain better performance than machine learning. Thus, the objectives of this study were: 1) to find the differences of average spectra of underground and aerial parts of *G. rigescens* Franch from 12 geographical origins; 2) to extract feature variables through two variable selection; 3) to compare the discriminant effects of machine learning and 1D-CNN using full spectra and feature variables; 4) to use spectral matrix as the input of 2D-CNN 5) to present the clustering effects of different layers in 2D-CNN by t-distributed stochastic neighbor embedding (t-SNE); 6) to visually display the spectral matrix pixels that had an high important impact on the discrimination results of 2D-CNN through saliency map.

## Materials and Methods

### Sample Preparation

The main medicinal component of *G. rigescens* Franch is located in the root, however recent studies have shown that the anti-inflammatory effect of the aerial parts of *G. rigescens* Franch is better than that of the underground parts (An et al., 2003). Therefore, both the aerial and underground parts of *G. rigescens* Franch were set as research objects in this study. Plants of *G. rigescens* Franch were collected from 12 different places in Yunnan and Guizhou province, China, as shown in Supplementary Table S1. Only samples from Houyan (geographical origin 3) were domesticated, while the rest were wild. Wild plants of *G. rigescens* Franch grew in natural populations, which were labeled by longitude and latitude. Wild plants of *G. rigescens* Franch are very precious and few in number. We collected eight plants from Puer, Yunnan (geographical origin 2), and ten plants from each of the remaining geographical origins. The plants were rinsed with deionized water to remove dust and soil. Then, aerial and underground parts were separated for each plant. They were put in an oven at 40°C for 5 h to remove the moisture. Afterwards, the aerial and underground parts of the same plant were ground separately at 60 Hz for 1 min. Finally, 0.15 g powders were pressed into a tablet with a diameter of about 15 mm in a pressure of 20 MPa for 30 s. Therefore, there were 236 tablets in total.

### Experimental Setup

**FIGURE 1:**
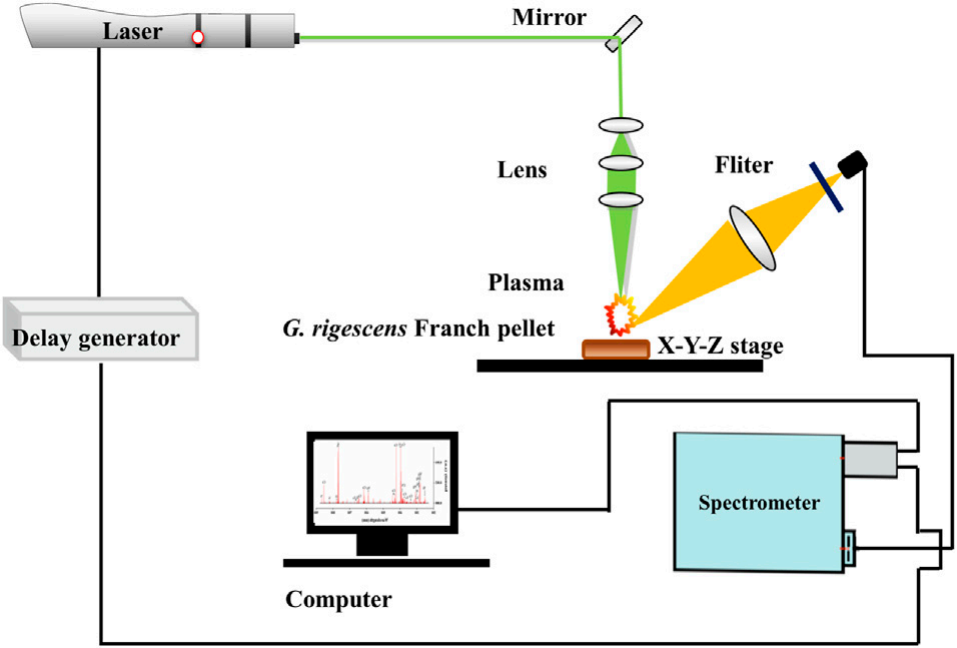
The schematic diagram of the laser-induced breakdown spectroscopy (LIBS) experiment.

The experiment was carried out with a self-assembled LIBS system, of which the detailed information could refer to this article (Peng et al., 2017). The schematic diagram is shown in Figure 1. The laser (532 nm) with a pulse duration of 8 ns was generated by Q-switched Nd:YAG pulsed laser (Vlite-200, Beamtech Optronics, Beijing, China). A self-designed optical system was used to direct the laser to the sample’s position. Right above the sample, a plano-convex lens (f = 100 mm) was fixed to focus the laser 2 mm below the surface of the sample. Under the action of laser ablation, the plasma was excited with an extremely short lifetime. The spectra were collected before the plasma disappeared using a spectrometer (ME5000, Andor, Belfast, UK), which could split a spectrum with a range from 229 to 878 nm with a resolution of 0.01 nm. Then the spectra were converted into electrical signals by an intensified charge coupled device (ICCD) camera and recorded in a computer. To prevent repeated ablations, an X-Y-Z motorized stage was applied to move the sample. The samples of *G. rigescens* Franch are not easy to be obtained. To reduce the impact of a small number of samples, 16 different positions in a tablet were ablated. Each position was ablated five times in succession and the five spectra were averaged. Therefore, there were 16 spectra for each tablet and 3,776 spectra in total. Delay time and gate width were the two important parameters for LIBS system, which were optimized as 1.5 and 10 µs respectively with reference to these articles (Shen et al., 2018; Liu et al., 2019) about plant samples. The energy of laser was set as 60 mJ.

### Data Preprocess

A wavelet transform was applied to eliminate random noise. The values of the wavelet function Daubechies and the decomposition level were optimized in the range of 3–10 according to the maximum SNR (signal-to-noise ratio). For aerial and underground parts’ spectra, the two parameters were optimized as (4,3) and (5,3) respectively. To reduce fluctuations from point to point (Bolger, 2000), area normalization method was used for each LIBS spectrum using the following equation:

$$X_{i}=\frac{x_{i}}{\sum_{i=1}^{n}x_{i}}$$ (1)

where $x_{i}$ is the i-th variable relative intensity measured by the instrument, n is 22015 which is the total number of LIBS spectral variables, $X_{i}$ is the relative intensity by area normalization. All the tablets of *G. rigescens* Franch for both the underground and aerial parts were randomly divided into the calibration set, validation set, and prediction set according to the ratio of 3:1:1. The number of spectra in the three corresponding datasets was 1,120, 384 and 384 respectively for both the underground and aerial parts.

### Variable Selection and Spectral Matrix

**FIGURE 2:**
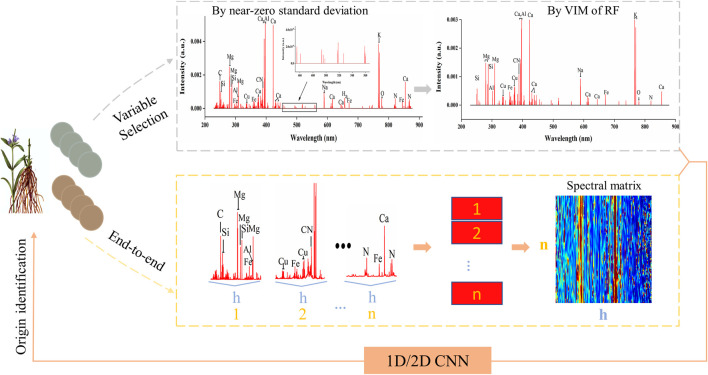
The geographical origin identification flowchart using convolutional neural network including variable selection and end-to-end learning based on spectral matrix.

In this study, variable selection and spectral matrix was used for 1D-CNN and 2D-CNN respectively. For variable selection, the data transmission including two consecutive variable selections was marked using grey dotted lines in Figure 2. The first variable selection was to remove the LIBS noise variables with near-zero standard deviation (Boucher et al., 2015). And the second variable selection was to reserve the feature variables by variable importance measurement (VIM). Noise signals have a relatively lower value of the standard deviation in LIBS spectra. Moreover, variables with low standard deviation also mean that the differentiation in all samples of them is little, which is not conducive to the discrimination by models. Therefore, variables with near-zero standard deviation were removed firstly in this study, which could also reduce the number of variables for the second selection to decrease the selection time.

Random forest (RF) is a tree based nonparametric ensemble learning method, which can effectively deal with high-dimensional variable problems for classification and regression (Strobl et al., 2009; Shi et al., 2019). The number of the trees had a significant impact on the classification accuracy, which was optimized in the range of 50–110 in this study. For each decision tree, about a third of the data do not participate in the growth of the tree, which are called the out of bag data (OOB). OOB can be used to evaluate the performance of the decision tree and calculate the prediction error rate of the model, which is called OOB error. After adding random noise to a variable, the larger the OOB error’s change is, the more important the corresponding variable is. The score of a variable importance is calculated with the following equation:

$$\text{Vim}=\frac{\sum OOBerror2-OOBerror1}{N}$$ (2)

Where Vim is the score based on the variable importance, *OOBerror2* is the OOB error after adding random noise and *OOBerror1* is the OOB error without random noise, *N* is the total number of decision trees. In this study, RF was used as a second variable selecting. Spectral variables were sorted by Vim, and 5% of the variables with the lower score were removed. This process was repeated 60 times. For each iteration, the number of decision trees was optimized in the range of 50–110. The feature variables were finally determined by the classification accuracy of the OOB.

For spectral matrix, the data transmission was marked using yellow dotted lines in Figure 2. Firstly, we cut a LIBS spectrum into *n* segments of equal length (*h* variables). Since there were 22,015 variables, *n* was the integer part of 22015/*h*. Then the *n* segments were recombined into a spectral matrix sequentially. In this study, *h* was selected as 110, 150 and 200 for a comparison analysis. As we all know, CNN is particularly suitable for image data processing relying on the two-dimensional and self-adaptive characteristics of the convolution kernel (Zhang et al., 2020). Therefore, we proposed a new form of LIBS data by recombining spectrum segments into a spectral matrix. On the other hand, this method could offer more spectral information to CNN and achieve the end-to-end training.

### Discriminant Analysis Method

#### Machine Learning

Linear discriminant analysis (LDA) is a classical linear classification algorithm, which was first proposed by Fisher (1936). The idea of LDA is projecting the samples onto a straight line, so that the projection points of similar samples are as close as possible. Then, samples from different categories can be distinguished.

K-nearest neighbor (KNN) is the simplest non-linear classification algorithm in machine learning. It calculates the distance among the samples and k nearest samples are regarded as one category (Chu et al., 2018). In this study, the optimal parameter k (3–20) was determined by the validation set’s discriminant accuracy.

Support vector machine (SVM) is a stable binary classification model (Vapnik and Chapelle, 2000). SVM divides the samples into two categories by the optimal hyperplane composed of support vector points. The SVM model has two characteristics. The first is to map low data to high-dimensional space through kernel function, and the second is to add penalty term in the optimal function to make SVM fault-tolerant to some extent. Kernel function parameter g determines the linearity of the hyperplane and the regularization parameter c determines the capacity of fault tolerance (Yu et al., 2016). In this study, the two parameters were optimized through grid searching in the range of 10^–8^–10^8^ and determined by classification accuracy of cross validation set.

#### Convolutional Neural Network

Deep learning method has been increasingly used in spectral data analysis (Kamilaris and Prenafeta-Boldú, 2018). Convolutional neural network (CNN) is one of the well-known deep learning structures for classification (Feng et al., 2019). AlexNet, an 8-layer convolutional neural network, won the Image Recognition Challenge based on ImageNet in 2012 with great advantage. Therefore, people considered adding more convolutional layers to reduce the error. However, in practice, the training error tended to increase rather than decrease after adding too many layers. To solve this problem, the residual network (ResNet) was proposed and won the first prize in the 2015 Image Recognition Challenge. AlexNet and ResNet were designed for image recognition. In this study, 1D-CNN1 and 1D-CNN2 with the network structures similar to AlexNet and ResNet were designed to identify LIBS spectra of *G. rigescens* Franch. By applying the same convolutional kernel in a spectrum, CNN could be used to identify important regions of the one-dimensional spectra (Acquarelli et al., 2017). At the same time, 2D-CNN with a simpler network structure than 1D-CNN1 and 1D-CNN2, was designed to identify spectral matrix.

**FIGURE 3:**
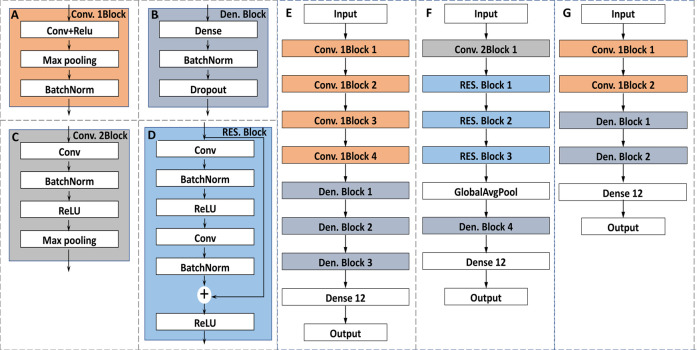
The architectures of the proposed classification models: **(A)** the architecture of the Convolution 1Block; **(B)** the architecture of the Dense Block; **(C)** the architecture of the Convolution 2Block; **(D)** the architecture of the Residual Block; **(E)** the architecture of the 1D-CNN1; **(F)** the architecture of the 1D-CNN2; **(G)** the architecture of the 2D-CNN.

1D-CNN1, similar to AlexNet, was mainly composed of two blocks including Convolution 1Block (Conv. 1Block) and Dense Block (Den. Block) as shown in Figures 3A,B. Batch normalization (BatchNorm) can improve the training speed and focus less on initialization (Wu et al., 2019). Pooling layer (Max pooling) was used to reduce the sensitivity of convolution to wavelength location. To prevent overfitting, dropout layer was added to randomly reduce the number of the neurons (Nie et al., 2019). The probability of deleting neurons was selected as 0.3 in this study. Others were some basic structures of CNN, such as convolutional layer (Conv) or dense layer (Dense). The activation function of them was rectified linear unit (ReLU) to improve the nonlinear learning ability of the network. The detailed architecture of the CNN1 is shown in Figure 3E. The numbers of kernels were 512, 128, 64 and 16 for Convs in Conv. 1Block 1, 2, 3 and 4, respectively. The size of kernel was three for Convs in four Conv. 1Blocks. The numbers of the neurons for Dense in Den. Block1, 2 and 3 were defined as 256, 64, 32, respectively.

1D-CNN2, similar to ResNet, was mainly composed of two blocks including Convolution 2Block (Conv. 2Block) and Residual Block (RES. Block) as shown in Figures 3C,D. The design of ResNet structure refers to the well-known ResNet for image classification (Nie et al., 2019). The main difference between CNN1 and CNN2 is the addition of a RES. Block, in which the input can be propagated forward more quickly through a cross-layer data path. The detailed architecture of the CNN2 is shown in Figure 3F. For Conv. 2Block1, the number and size of the kernel was 64 and 3, respectively. For each RES. Block, the kernel size of Conv and the number were the important parameters with the values of (64, 2), (128, 2) and (256, 2) for RES. Block 1 2 3, respectively. For Den. Block 4, the number of the neurons was defined as 128.

The detailed architecture of the 2D-CNN is shown in Figure 3G. The number of kernels was 64 and 16 for Convs in Conv. 1Block 1 and 2, respectively. The corresponding kernel size was 7 and 3. The larger kernel size can capture more spectral matrix features at once. The number of neurons in Den. Block 1 and Den. Block 2 were defined as 256 and 64, respectively.

Different learning rates and thresholds were set to train the CNN based on stochastic gradient descent (SGD). In each training process, the training ended when the classification accuracy of the validation set reached the threshold, or when the number of iterations reached 1,000. In the process of piecewise training, the learning rate decreased gradually and the corresponding thresholds gradually increased until the model converged. Taking 2D-CNN (*h* = 150) based on underground parts as an example, the learning rates were set as 0.1, 0.05, 0.01 and 0.005, respectively, and the corresponding thresholds were set as 0.6, 0.75, 0.92, and 0.96. The accuracy of the validation set finally converged to 0.95.

### Model Evaluation and Visualization

Discriminant accuracy was used to evaluate the performance of models in this study. Discriminant accuracy was defined as the ratio of the number of correctly discriminated spectra to the total number of spectra.

A confusion matrix was conducted to analyze the effect of the optimal model further. The difference between the prediction results and actual measurements for each geographical origin could be visually observed. The confusion matrix’s vertical axis represented the real class, and the horizontal axis represented the prediction class.

T-distributed stochastic neighbor embedding (t-SNE) was used to visualize the clustering process of the extracted features from the CNN. It could realize the nonlinear dimension reduction of high-dimensional spectra data (Van Der Maaten and Hinton, 2008). The Gaussian distribution’s perplexity was defined as 30, and the initial dimensions of PCA were defined as 12 in t-SNE. For the output data with three dimensions, the dimension representing sample number was retained and the data from other two dimensions were averaged (Zhang et al., 2020).

Saliency map can obtain the weight of pixels through the back-propagation algorithm (Liu et al., 2011). The larger the weight, the greater the influence of the corresponding pixels on the model. Therefore, every spectral matrix has a corresponding saliency map. In this study, the weights of spectral matrix in the prediction set from the same origin were averaged to represent the origin’s saliency map. Because the spectral matrix was obtained by spectrum segmentation and recombination, we could transform the spectral matrix back to a spectrum using the same rule. So, the spectral wavelengths could correspond to the pixels one by one. In this way, we could extract the important wavelengths according to the weight of the pixels in a saliency map.

### Software and Hardware

The machine learning algorithms were run on Matlab R2014b (The MathWorks, Natick, MA, USA). The software was installed on a Windows7 Desktop with Intel Xeon E5-2620 and 64 GB RAM. Convolutional Neural Network was deployed on the framework of Apache MXNet1.4.0 in another computer of Ubuntu Desktop with GTX1080Ti (NVIDIA, California, USA) and 48 GB RAM.

## Results

### Average Spectral Analysis

**FIGURE 4:**
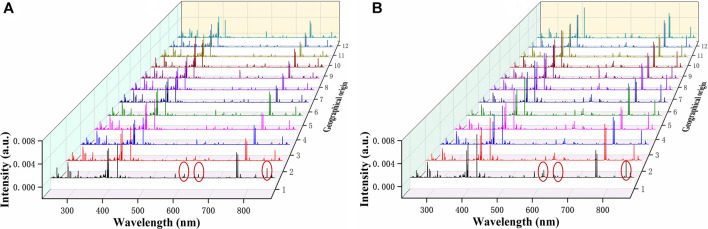
The average LIBS spectra of *G. rigescens* Franch from 12 geographical origins: **(A)** underground parts and **(B)** aerial parts.

The average LIBS spectra of *G. rigescens* Franch from 12 geographical origins is shown in Figure 4. For underground and aerial parts, the excitation wavelengths of the spectral lines were almost the same, as they all originated from the *G. rigescens* Franch’s plants and had similar elements. However, there were some slight differences in spectral intensity in some spectral lines (circled in red), which represented for Ca (612.30, 616.38, 854.29 nm) and H (656.28 nm) according to the National Institute of Standards and Technology (NIST) database. The relative intensity of Ca spectral lines in aerial parts were higher than that in underground parts, which was consistent with the findings in this article (Dong et al., 2015). The spectral lines of H in aerial parts were barely visible, but they could be seen in the spectra of underground parts. This might be related to the medicinal ingredients in the roots of loganic acid (C_16_H_24_O_10_) and gentiopicroside (C_16_H_20_O_9_). It was impossible to distinguish the geographical origin by LIBS spectrum. Therefore, further analysis was needed by identification models.

### Variable Selection

For underground parts, the standard deviation of all LIBS variables in 2048 spectra were calculated and arranged in order from smallest to largest (see Supplementary Figure S1A). By human observation, the standard deviation of the variables before the red dot were close to 0. Therefore, a total of 2016 variables after red point were retained. For aerial parts (see Supplementary Figure S1B), the result was similar to the underground parts and 2016 variables were left.

**FIGURE 5:**
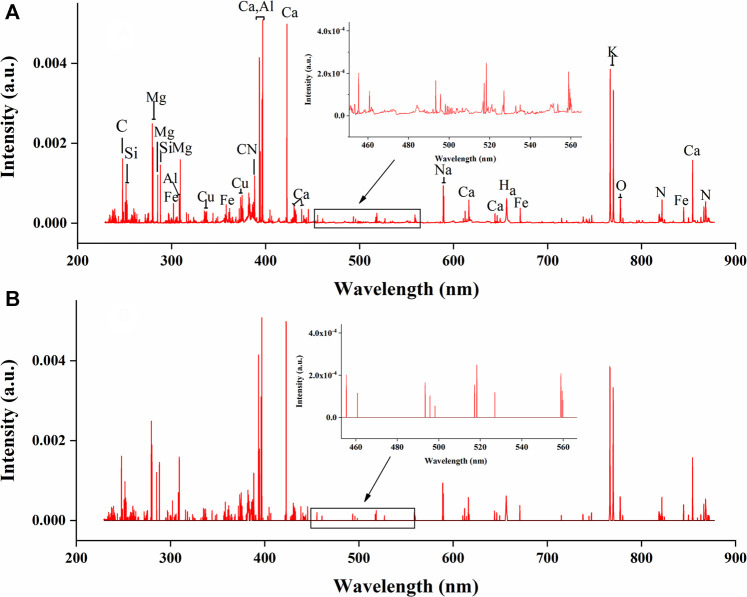
The average LIBS spectra for underground parts of *G. rigescens* Franch with **(A)** original variables and **(B)** reserved variables by standard deviation. Note: The variables with near-zero standard deviation were set as “0” and colored black for better comparison.

The LIBS spectra for underground parts of *G. rigescens* Franch before and after variable removing is shown in Figures 5A,B. In Figure 5B, the variables with near-zero standard deviation were set as “0” and colored black for better comparison. We could find that most of the signals were retained, including signals standing for nutrient element and signals with relatively low intensity. From the detail diagram, it could be found that the noise signals with near-zero intensity were effectively removed. In the comparison of Supplementary Figures S2A,B, for aerial parts of *G. rigescens* Franch, the same results could be observed. Therefore, removing the variables with near-zero standard deviation was an efficient method to eliminate noise of LIBS spectra. In this study, through this method, the number of variables decreased by 90.8% from 22015 to 2016 for both underground and aerial parts of *G. rigescens* Franch.

Although the noise signals were eliminated, the variables that could effectively represent the origin information needed to be further selected. RF is a supervised classification model, which can screen out the variables highly related to the geographical origins by VIM. Supplementary Figure S3 shows the change in the accuracy of the OOB as the number of iterations increases. It could be seen that as the number of iterations increased, the accuracy increased first and then decreased. The optimal iteration number was 35 and 38 for underground and aerial parts respectively with the corresponding accuracy of 96 and 93%. Therefore, for underground and aerial parts, when the number of iterations reached 35 and 38 respectively, the remaining variables were retained as the result of the second variable selection.

**FIGURE 6:**
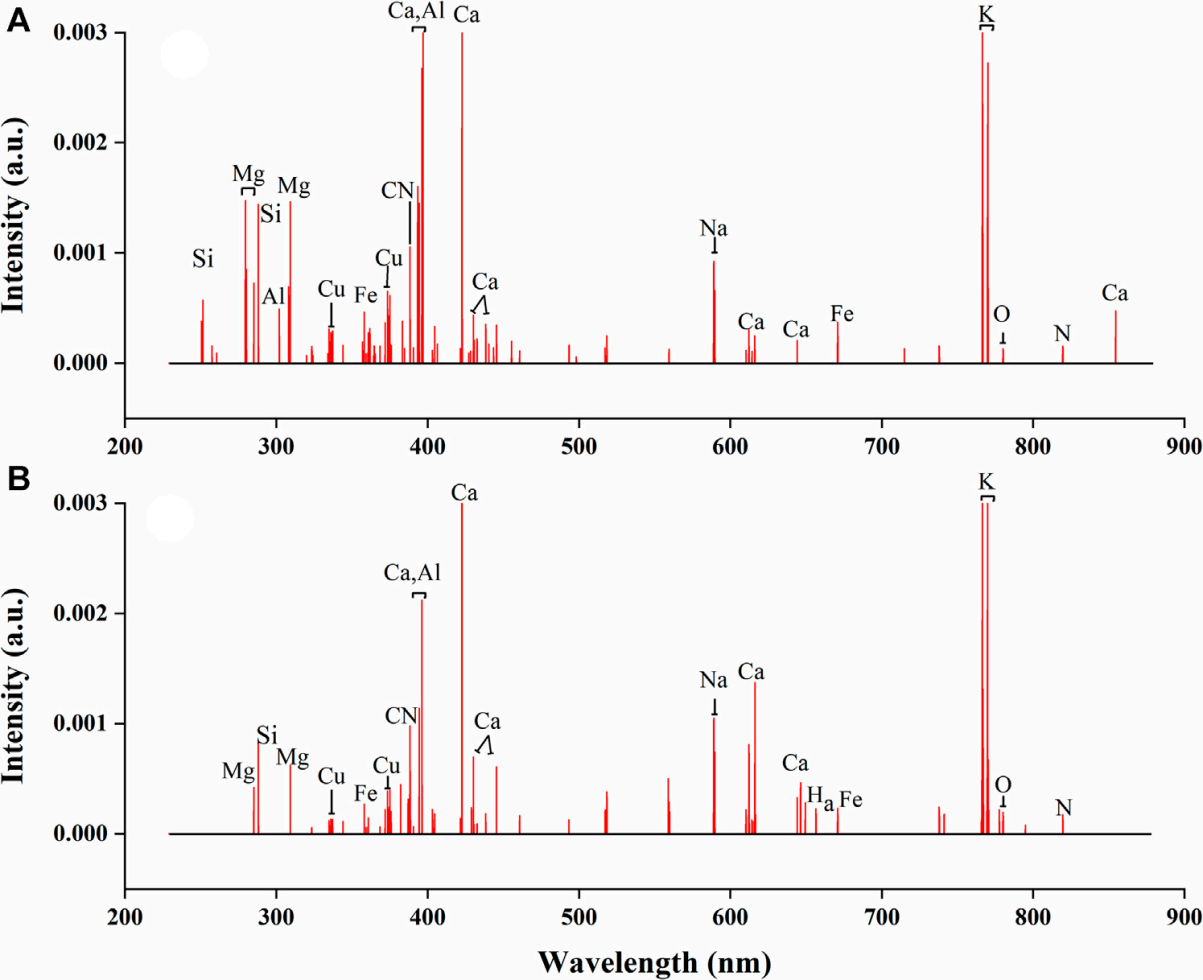
The selected variables by VIM using RF in LIBS spectra for **(A)** underground parts and **(B)** aerial parts of *G. rigescens* Franch.

The variables selected by VIM of RF are shown in Figure 6. The number of reserved variables were 325 and 277 for underground and aerial parts respectively, which were further reduced by 83.8 and 86.3% based on the first variable selection. Most of the preserved spectral lines represented for nutritive elements, indicating that the nutritive element content of *G. rigescens* Franch was closely related to its geographical origin. The results showed the effectiveness of VIM of RF in selecting important variables for LIBS spectra.

### Discriminant Analysis Using Full Spectra and Selected Variables

Table 1 shows the results of discriminant models based on underground parts of *G. rigescens* Franch using full spectra and selected variables. With the gradual selecting of variables, the accuracy of the validation set and prediction set of most discriminant models displayed an increasing trend, which indicated the effectiveness of the variable selection. For linear machine learning of LDA, with the gradual selecting of variables, the accuracy in the prediction set was 59.90, 66.41 and 74.48%, respectively. Non-linear machine learning (KNN and SVM) obtained better performance than LDA. For KNN, with the gradual selecting of variables, the accuracy in the prediction was 82.03, 82.03 and 88.02%, respectively. For SVM, the corresponding accuracy was 91.93, 89.58 and 88.02%, respectively. LDA can only extract linear relationships of data (Li et al., 2018). Therefore, LDA usually has worse performance than other non-linear machine learning algorithms (Benos et al., 2021). SVM obtained better performance than KNN. This is because the advantages of SVM are structural risk minimization rather than the empirical risk minimization (Belayneh et al., 2014).

**TABLE 1:** The results of discriminant models based on underground parts of *G. rigescens* Franch using full spectra and selected variables.

Variables selection method (number of variables	Model	Cal^1^(%)	Val^2^(%)	Pre^3^(%)
Full variables (22015	LDA	73.35	65.89	59.90
	KNN	89.46	68.75	82.03
	SVM	100.00	86.72	91.93
	1D-CNN1	100.00	8.33	8.33
	1D-CNN2	100.00	8.33	8.33
First variable selection (2016	LDA	75.95	74.22	66.41
	KNN	88.93	68.75	82.03
	SVM	100.00	89.06	89.58
	1D-CNN1	100.00	92.19	89.32
	1D-CNN2	100.00	90.36	88.54
Second variable selection (325	LDA	83.12	79.17	74.48
	KNN	90.18	73.44	85.94
	SVM	100.00	90.63	88.02
	1D-CNN1	100.00	92.45	92.19
	1D-CNN2	100.00	87.24	89.84

^1 2 3^. Cal, Val and Pre are assigned respectively as the discriminant accuracy of calibration set, validation set, and prediction set.

For 1D-CNN, the results based on full spectra were overfitting, because the number (22014) of variables was so much higher than the number (1,120) of spectra that the accuracy of the validation set was always around 0.08% even though the accuracy of the calibration set reached 100%. Thus, variables selection was of great significant to the better training of 1D-CNN models. After the second variable selection, the accuracy of the prediction set was the highest with the value of 92.19% based on 1D-CNN1. This indicated the effectiveness of variable selection. The running time of SVM and 1D-CNN1 is shown in Supplementary Figure S4A. With the gradual variable selection, the training time of SVM and 1D-CNN1 became less and less with the value of 312.2 and 161.3 s respectively in the end. Therefore, the method of variable selection in this study could not only reduce the training time of models but also improve the discriminant accuracy. On the whole, 1D-CNN1 was the optimal model for the geographical origin identification of underground parts of *G. rigescens* Franch.

Supplementary Table S2 shows the results of discriminant models based on aerial parts of *G. rigescens* Franch. The same results with above could be found that with the gradual selection of variables, the accuracy of the validation set and prediction set of most discriminant models displayed an increasing trend. In brief, after the second variable selection, 1D-CNN1 obtained the highest accuracy in the prediction set among the five models with the value of 94.01%. The running time of SVM and 1D-CNN1 is shown in Supplementary Figure S4B. After the second selection of variables, the running time of SVM and 1D-CNN1 was 269.4 s and 44.7 s, respectively. Thus, 1D-CNN1 was the optimal model for the geographical origin identification of *G. rigescens* Franch for both the underground and aerial parts.

### Discriminant Analysis Using Spectral Matrix

Table 2 shows the results of 2D-CNN with the input of spectral matrix. *H* was selected as 110, 150 and 200 for a comparison analysis. For underground parts, as the value of *h* increased, the accuracy in the prediction set was 93.49, 94.01 and 92.97%, respectively. The corresponding accuracy in the validation set was 95.57, 95.05 and 95.57%, respectively. This indicated that the 2D-CNN with the input of spectral matrix had a good generalization ability. Besides, all the results were better than the optimal result based on 1D-CNN1. And 2D-CNN had a much simpler network structure than 1D-CNN1. These indicated the effectiveness of changing the LIBS spectrum into spectral matrix by spectrum segmentation and recombination. At the same time, 2D-CNN combined with spectral matrix could achieve the end-to-end training. For aerial parts, the optimal value of *h* was also 150. The corresponding accuracy in the prediction set was 94.01%, which was the same as the optimal results by 1D-CNN1. However, the accuracy in the validation set was 93.23%, which was 4% higher than that of 1D-CNN1. On the whole, 2D-CNN combined with spectral matrix could capture the characteristics of geographical origin more effectively than 1D-CNN1.

**TABLE 2:** The results of 2D-CNN with the input of spectral matrix for underground and aerial parts of *G. rigescens* Franch.

Sample	*H*	Cal^1^(%)	Val^2^(%)	Pre^3^(%)
underground parts	110	100.00	95.57	93.49
	150	100.00	95.05	94.01
	200	100.00	95.57	92.97
aerial parts	110	100.00	93.23	92.71
	150	100.00	93.23	94.01
	200	100.00	96.35	92.45

**FIGURE 7:**
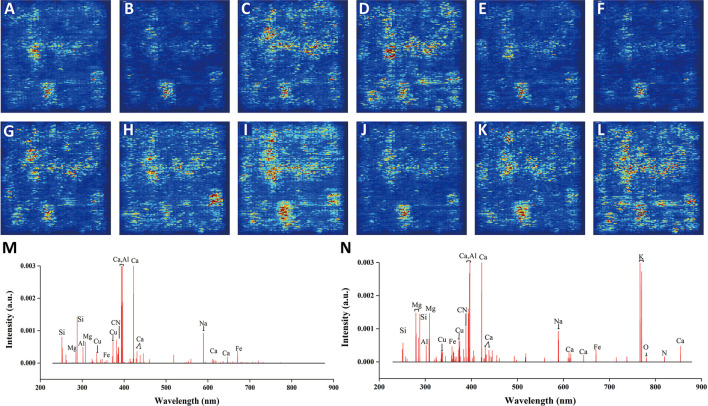
The average saliency map of each geographical origin based on 2D-CNN for underground parts of *G. rigescens* Franch. **(A–L)** represent 12 different geographical origins. **(M, N)** represent the important wavelengths selected by 2D-CNN and VIM of RF, respectively.

Convolution kernel is especially suitable for processing image data due to the two-dimensional and self-adaptive characteristics (Yan et al., 2021). Therefore, the input of spectral matrix could be better processed by 2D-CNN. For a better interpretation of good results by 2D-CNN, we extracted the wavelengths that had a higher influence on the model results by saliency map (see Figure 7M). Most of the wavelengths represented for nutritive elements, indicating the reasonability of the discrimination by 2D-CNN to some extent. At the same time, these wavelengths had certain similarities with the wavelengths (see Figure 7N) selected by VIM of RF. This further confirmed the rationality by using spectral matrix. The average saliency map of each geographical origin based on 2D-CNN is shown in Figures 7A–L. The darker the color, the greater the influence of the corresponding pixels on the results of the model. It could be seen that there was a certain overlap of saliency pixels from different geographical origins, because the spectra that made up the image were from the same TCM called *G. rigescens* Franch. There were also some differences in color intensity and distribution, representing the differences in different geographical origins, which was helpful for the discrimination of 2D-CNN.

### Confusion Matrix Analysis

**FIGURE 8:**
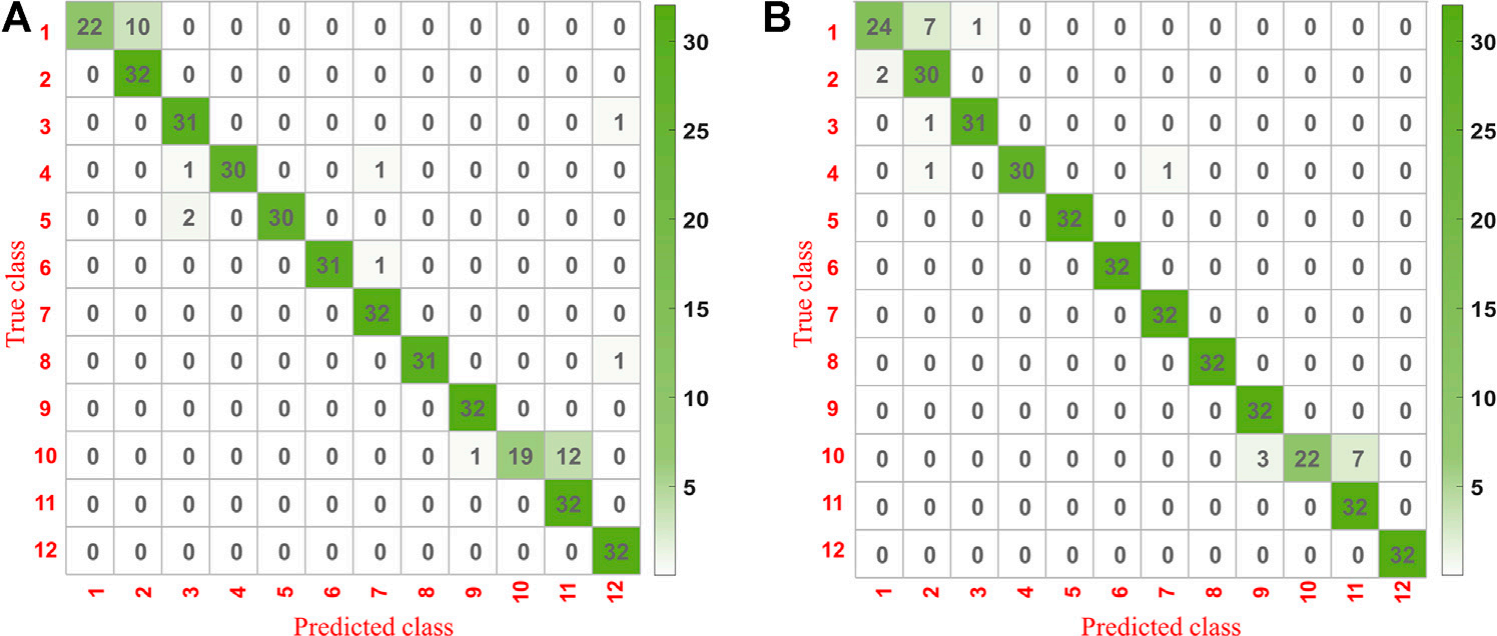
The confusion matrix for the prediction set of underground parts of *G. rigescens* Franch based on **(A)** 1D-CNN1 after the second variable selection and **(B)** 2D-CNN with the input of spectral matrix.

The confusion matrixes for the prediction set of underground parts of *G. rigescens* Franch based on 1D-CNN1 after the second variable selection and 2D-CNN are shown in Figures 8A,B. The bigger the value was, the darker the color presented. The value on the diagonal represented the number of samples that were correctly classified. It could be seen that all the values on the diagonal based on 2D-CNN were higher than that based on 1D-CNN1. This indicated that 2D-CNN with the input of spectral matrix was more appropriate in the discriminant analysis for LIBS spectra. For 2D-CNN, the misclassification rate of geographical origin 1 and 10 was a litter higher. The same phenomenon could be seen for 1D-CNN1. Therefore, special attention should be paid when identifying samples from geographical origin 1 and 10 in practical application. As for the remaining geographical origins, at most two spectra from one area could not be correctly identified. The results showed that 2D-CNN with the input of spectral matrix combined with LIBS technology could realize the geographical origin identification of *G. rigescens* Franch.

### Cluster Visualization of 2D-CNN.

**FIGURE 9:**
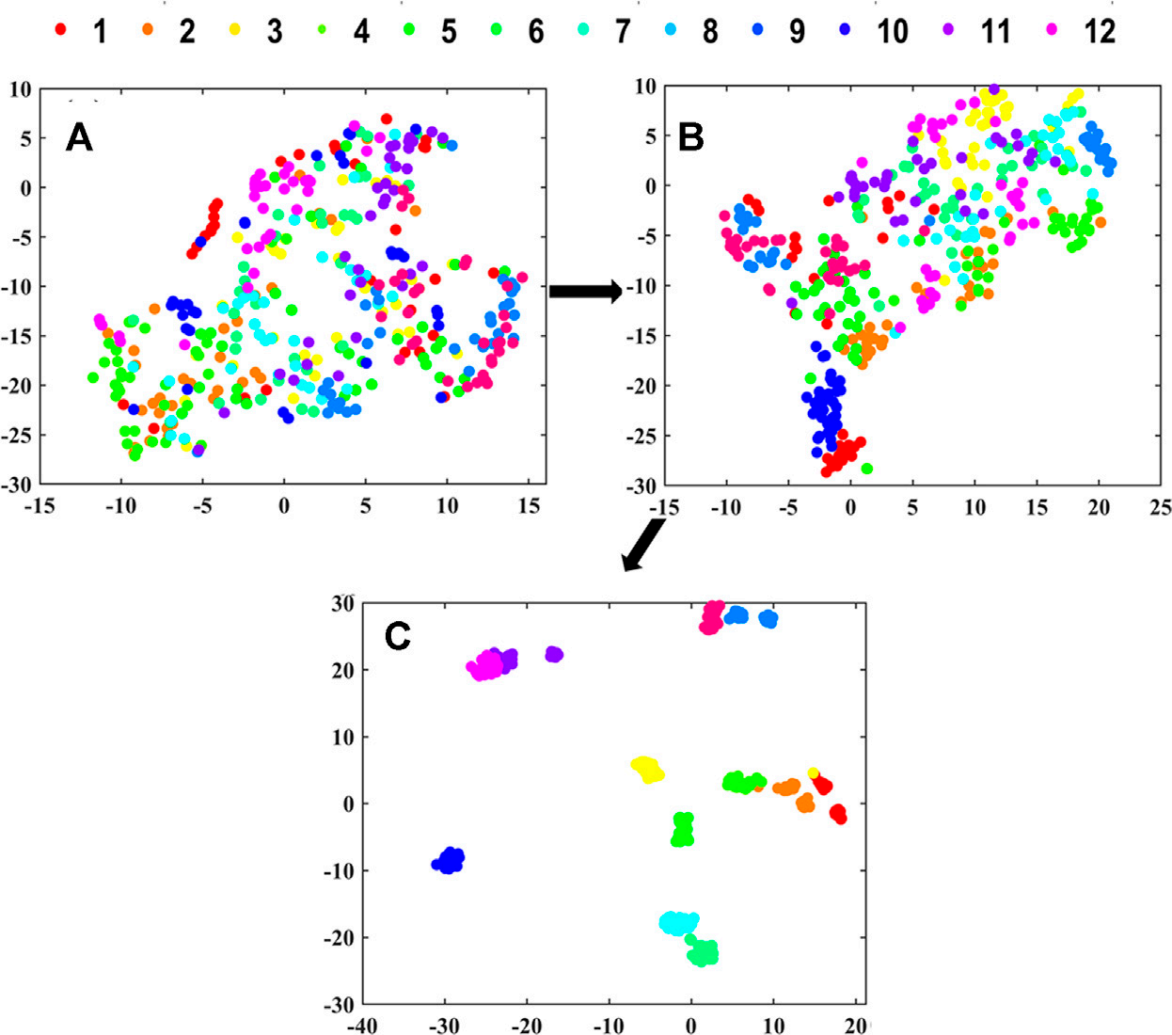
The clustering visualization in layers of **(A)** Conv. 1Block1, **(B)** Conv. 1Block2 and **(C)** Dense 12 in 2D-CNN for underground parts by t-SNE.

The clustering visualization in layers of 2D-CNN for underground parts is shown in Figure 9. For the shallower layers in 2D-CNN including Conv. 1Block1 and Conv. 1Block2 (see Figures 9A,B), the boundaries between different geographical origins were not clear, indicating that these layers have not been able to extract effective features representing different geographical origins. And for the deeper layers of Dense 12 (see Figure 9C), there were obvious boundaries between different geographical origins, indicating that deeper layers in 2D-CNN had learned the effective information related to geographical origin.

### The Advantages and Disadvantages of 2D-CNN Based on Spectral Matrix

For the advantages, taking the underground parts of *G. rigescens* as an example (see Table 1), 1D-CNN with the input of full spectra showed over-fitting phenomenon. What’s more, the optimal accuracy obtained by 1D-CNN coupled with variable selection was still 2% lower than that of 2D-CNN (from 92.19 to 94.01%). Therefore, 2D-CNN based on spectral matrix could restrain over-fitting phenomenon, improve accuracy and realize end-to-end training. When *h* (see Figure 2) changed from 110 to 200, the accuracy in the prediction set did not change much (see Table 2), which indicated that our method of transforming the single spectrum to spectral matrix had a good stability. Our method could provide a new idea for processing spectral data with high variables.

For the disadvantages, when *h* (see Figure 2) could not be divided exactly by the number of spectral variables, we had to directly discard the rest variables that couldn’t form a complete matrix. However, the discarded variables might be highly relevant to the geographical origin. In Figure 7M, we could find that some important variables after 700 nm were abandoned by our method compared with that in Figure 7N. Subsequently, we will consider weighting variables and the variables with low weights can be discarded first to preserve as many important variables as possible.

## Conclusion

Laser-induced breakdown spectroscopy combined with CNN could be used to achieve the fast geographical origin identification of *G. rigescens* Franch. 1D-CNN coupled with variable selection could improve the accuracy and reduce the training time effectively. 2D-CNN with the input of spectral matrix could improve the accuracy further. The optimal accuracy in the prediction set for underground and aerial parts of *G. rigescens* Franch was the same with the value of 94.01%. The method of 1D-CNN coupled with variable selection can provide some support for LIBS portable instrument development. The method of 2D-CNN combined with spectral matrix can offer some reference for the discriminant analysis of LIBS spectra with too many variables. At the same time, it can achieve the end-to-end learning of CNN. In the future, large number of samples should be prepared to improve the generalization ability of the CNN model further.

## Data Availability

The raw data supporting the conclusion of this article will be made available by the authors, without undue reservation.
