# Supplementary material for: Application of Laser-Induced Breakdown Spectroscopy Coupled With Spectral Matrix and Convolutional Neural Network for Identifying Geographical Origins of Gentiana rigescens Franch
Source: Front Artif Intell. 2021 Dec 10;4:735533. doi: 10.3389/frai.2021.735533 (PMC8703168; doi:10.3389/frai.2021.735533)
Supplement: Supplementary file 1 [file Table1.docx]

**Supplementary Table S1.** The geographical origin information of *G. rigescens* Franch

| **No.** | **Geographical origins** | **Longitude** | **Latitude** | **Number of Plants** |
| --- | --- | --- | --- | --- |
| 1 | Chuxiong, Yunnan | 101°20′27″ | 24°37′17″ | 10 |
| 2 | Puer, Yunnan | 101°11′ | 22°88′ | 8 |
| 3 | Houyan, Yunnan | 100°19′07″ | 24°18′17″ | 10 |
| 4 | Bijie, Guizhou | 105°16′11″ | 27°18′11″ | 10 |
| 5 | Liupanshui, Guizhou | 104°32′46″ | 26°23′09″ | 10 |
| 6 | Xuanwei, Yunnan | 104°26′11″ | 26°28′49″ | 10 |
| 7 | Malong, Yunnan | 103°39′20″ | 25°20′40″ | 10 |
| 8 | Dali, Yunnan | 100°07′38″ | 25°40′51″ | 10 |
| 9 | Xinping, Yunnan | 101°56′04″ | 24°01′20″ | 10 |
| 10 | Heqing, Yunnan | 100°17′52″ | 26°32′57″ | 10 |
| 11 | Kunming, Yunnan | 102°53′34" | 24°45′18″ | 10 |
| 12 | Dieshuitou, Yunnan | 99°50′25″ | 24°11′15″ | 10 |
