# Supplementary figures and images for: Application of Laser-Induced Breakdown Spectroscopy Coupled With Spectral Matrix and Convolutional Neural Network for Identifying Geographical Origins of Gentiana rigescens Franch
Source: Front Artif Intell. 2021 Dec 10;4:735533. doi: 10.3389/frai.2021.735533 (PMC8703168; doi:10.3389/frai.2021.735533)

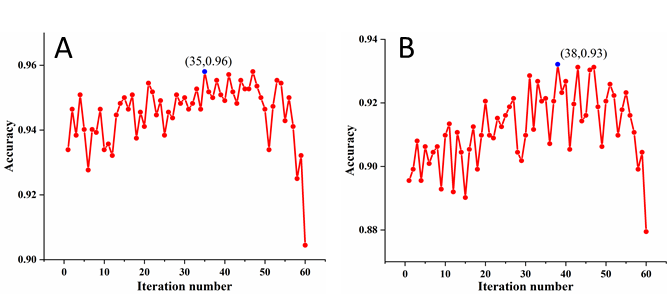

Supplement: Supplementary file 2 [file Image3.tif]

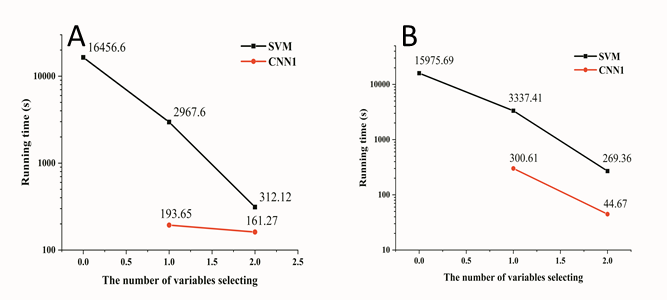

Supplement: Supplementary file 3 [file Image4.tif]

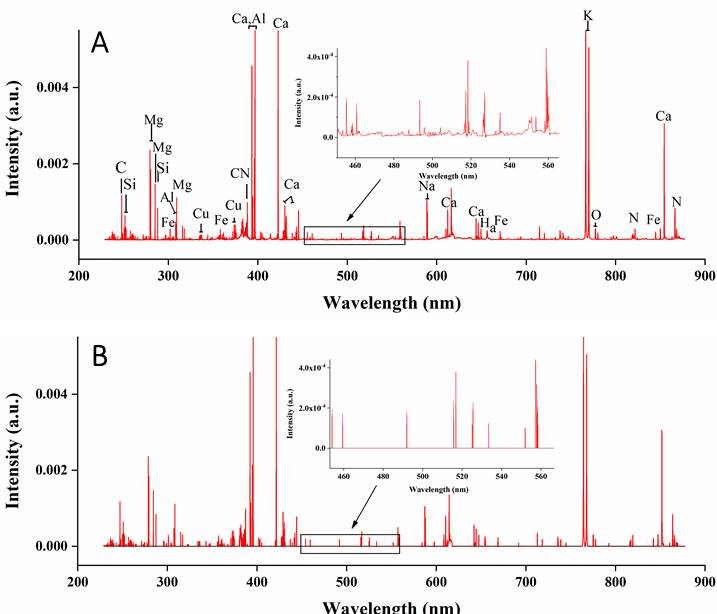

Supplement: Supplementary file 4 [file Image2.tif]

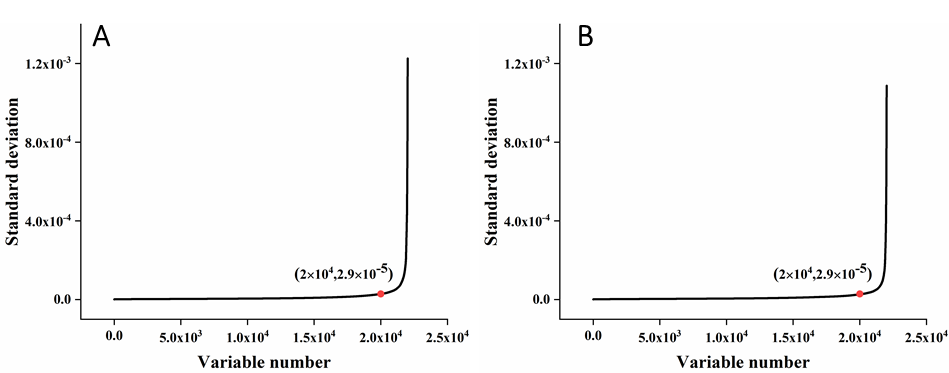

Supplement: Supplementary file 5 [file Image1.tif]
