# Supplementary material for: Application of Laser-Induced Breakdown Spectroscopy Coupled With Spectral Matrix and Convolutional Neural Network for Identifying Geographical Origins of Gentiana rigescens Franch
Source: Front Artif Intell. 2021 Dec 10;4:735533. doi: 10.3389/frai.2021.735533 (PMC8703168; doi:10.3389/frai.2021.735533)
Supplement: Supplementary file 6 [file Table2.docx]

| **Supplementary Table S2.** The results of discriminant models based on aerial parts of *G. rigescens* Franch using full spectra and selected variables | | | | |
| --- | --- | --- | --- | --- |
| **Variables selection method**  **(number of variables)** | **Model** | **Cal1(%)** | **Val2(%)** | **Pre3(%)** |
| Full variables  (22015) | LDA | 72.27 | 51.82 | 64.06 |
|  | KNN | 88.30 | 65.63 | 61.20 |
|  | SVM | 100 | 89.32 | 92.97 |
|  | CNN1 | 100 | 8.33 | 8.33 |
|  | CNN2 | 100 | 8.33 | 8.33 |
| First variable selection  (2016) | LDA | 77.08 | 63.28 | 65.1 |
|  | KNN | 80.00 | 63.02 | 65.36 |
|  | SVM | 99.64 | 89.06 | 89.58 |
|  | CNN1 | 100 | 88.02 | 88.28 |
|  | CNN2 | 100 | 84.90 | 88.02 |
| Second variable selection  (277) | LDA | 83.03 | 70.05 | 72.66 |
|  | KNN | 92.05 | 78.13 | 79.69 |
|  | SVM | 100 | 88.54 | 90.63 |
|  | **CNN1** | **100** | **89.32** | **94.01** |
|  | CNN2 | 100 | 87.50 | 89.84 |

^1 2 3.^ Cal, Val and Pre are assigned respectively as the discriminant accuracy of calibration set, validation set, and prediction set.
